# Tracking Performance Limits Using Multi‐Timescale Maximal Mean Power Ratios

**DOI:** 10.1002/ejsc.70179

**Published:** 2026-05-18

**Authors:** Andrea Zignoli, Andrea Giorgi, Filip Kolodziej, Borja Martinez‐Gonzalez, Peter Leo, Paul B. Laursen

**Affiliations:** ^1^ Department of Neurosciences Biomedicine and Movement Sciences University of Verona Verona Italy; ^2^ Department of Industrial Engineering University of Trento Trento Italy; ^3^ Athletica Inc. Revelstoke British Columbia Canada; ^4^ Medical and Performance Staff VF Group ‐ Bardiani CSF—Faizanè Professional Cycling Team Bibbiano Italy; ^5^ Department of Internal Medicine Specialist Medicine and Rehabilitation Azienda ASL Toscana Sud‐Est Siena Italy; ^6^ School of Health Sciences and Social Work Griffith University Gold Coast Australia; ^7^ Kent and Medway Medical School University of Kent Canterbury UK; ^8^ Department of Sports Science University of Innsbruck Innsbruck Austria; ^9^ UniSA Allied Health & Human Performance University of South Australia Adelaide Australia; ^10^ Sports Performance and Athlete Development Environments (SPADE) University of Agder Kristiansand Norway; ^11^ Sports Performance Research Institute New Zealand (SPRINZ) AUT University Auckland New Zealand

**Keywords:** endurance performance, mathematical modeling, professional cyclists, record power profile

## Abstract

This study introduces a multi‐timescale mechanical model to quantify proximity to performance limits during endurance exercise. The model represents power output using a set of rolling averages, each associated with a characteristic time constant, and identifies the dominant component as the one approaching its historical maximum at any given time. To demonstrate this framework, real‐world data were collected from 21 male professional cyclists during an 11‐day training camp. Data from the first 10 days were used to construct individual maximal mean power (MMP) profiles across multiple time scales. On the final day, cyclists completed a fatiguing protocol (∼2000 kJ of work) followed by 3‐min and 12‐min maximal time trials. During exercise, the ratio between each exponentially weighted component and its corresponding historical maximum was computed, and the maximum ratio was used to track proximity to performance limits. At the end of the time trials, this ratio reached 98.6% (94.3%–101%) and 101% (98.5%–103%) for the 3‐min and 12‐min efforts, respectively (median and interquartile range), indicating convergence toward maximal performance capacity. Notably, in both trials the dominant component corresponded to a slower time scale (∼1 h), rather than to components matching the nominal duration of the efforts. These findings suggest that performance limits emerge from the interaction of multiple time scales and are not solely dictated by the duration or intensity of the task. This framework extends the traditional use of MMP from a post hoc descriptive tool to a real‐time dynamical measure of performance capacity.

## Highlights

- A model based on rolling averages of mechanical power output across multiple time scales is introduced to continuously quantify the margin between current exercise intensity and historically observed performance limits during stochastic exercise.
- Maximum performance was defined as the point at which the maximal ratio between current and historical responses across all considered time scales reached 100%, indicating that at least one component of the system had reached its previously observed maximum.
- The model was evaluated using data from 21 professional cyclists performing 3‐ and 12‐min time trials following a prolonged fatiguing protocol designed to induce prior accumulation of work.
- The model describes dynamic changes in performance limits during exercise by accounting for the cumulative effects of prior intensity and duration, allowing identification of the time scale governing progression toward or away from their performance limit.

## 1 Introduction

The human body can be conceptualized as a mechanical energy‐production system that works with variable efficiency. The ability to sustain energy output over prolonged durations, despite fatigue, overheating, or declining efficiency, is the foundation of endurance sport performance (McCormick et al. 2015).

In cycling, this system is typically observed through its external mechanical output, namely power production (W), which provides a measurable representation of performance over time (Jobson et al. 2009). Power profiles describe how power output varies across different exercise durations (Leo et al. 2022), using rolling averages to capture performance fluctuations across time scales. The highest values of these rolling averages define the historical maximal mean power (MMP), which summarizes an athlete's performance limits across durations (Pinot and Grappe 2011; Quod et al. 2010).

Although MMP is widely used to characterize performance retrospectively (see e.g., (Mateo‐March et al. 2022; Valenzuela et al. 2023; Valenzuela et al. 2023)), it does not provide a mechanism to track how an athlete's current output relates to their maximal historical capacity *during* exercise. Here, we propose a multi‐timescale framework to quantify the relationship between current power output and historical MMP in real time. By representing power output through a set of rolling averages, each associated with a characteristic time constant, the model identifies the component most closely approaching its historical maximum at any given moment. This dominant component is then used as an indicator of proximity to the athlete's performance limits. This formulation extends the traditional use of MMP from a post hoc descriptive tool to a real‐time dynamical representation of performance state during exercise (Zignoli and Whitehurst 2025).

## 2 Methods

### 2.1 Training Data

The data to validate the model presented here was collected on a cohort of 21 male professional cyclists (age 23 ± 4; height 176.7 ± 5.2 cm; body mass 66.3 ± 4.4 kg), who participated in a training camp from December 6th to 16th, 2023 and served as study participants. Ethical approval for this study was obtained from the Regional Health Unit Authority Ethics Committee (code: AGBMG23). Before the training camp, all cyclists were fully informed of the study objectives and provided written informed consent to share their data for research purposes. All participants were members of the same professional cycling team, ranked among the top 25 World Tour teams by the Union Cycliste Internationale in 2023. Each training session was recorded using personal cycling computers (Bryton S800, Bryton 86 Inc., Taipei City, Taiwan) and power meters (Favero Assioma Duo, Favero Electronics srl., 84 Arcade, TV, Italy) mounted on the cyclists' bikes. Data, including session duration and power output (W), were collected at a sampling frequency of 1 Hz.

**FIGURE 1:**
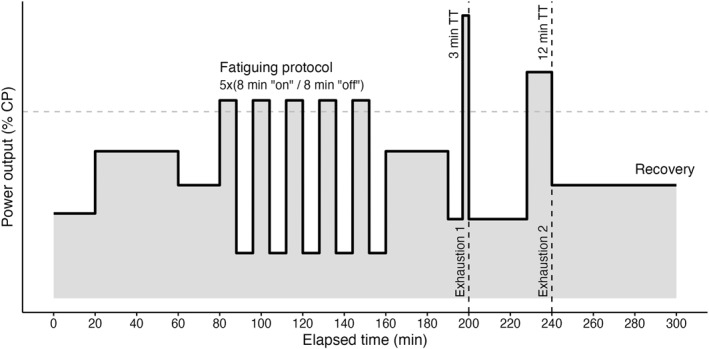
Protocol executed by the cyclists on the final day of the training camp. Following a 20‐min warm‐up, participants completed a “fatiguing protocol,” after which they performed a “time‐trial” protocol. The time‐trial protocol consisted of two maximal efforts lasting 3‐ and 12‐min, separated by 40‐min of active recovery.

On the first day of the camp, participants completed a test to assess their CP, as described in Spragg et al. (2024). The CP values obtained from this test were used to prescribe subsequent training sessions for each cyclist. On the final day, all cyclists completed a structured “fatiguing” protocol (Spragg et al. 2024) (Figure 1).

The fatiguing protocol consisted of ∼2000 kJ of work, beginning with 20 min at < 70% of individual CP, followed by five 8‐min intervals at 105%–110% of CP, interspersed with 8‐min recovery periods. During recovery, participants maintained a rating of perceived exertion (RPE) of 2 out of 10, corresponding to “light exertion”. Following the protocol, the cyclists performed two maximal time trials of 3 and 12 min, separated by 40 min of active recovery. Participants were instructed to complete the trials at maximal effort until the end of their sustainable performance capacity, while maintaining a cadence of 80–100 revolutions per minute and using a self‐selected pacing strategy. To facilitate optimal pacing, participants were permitted to monitor real‐time power output on their cycling computers.

### 2.2 Power Profiling

The MMP method (Pinot and Grappe 2011; Quod et al. 2010) does not impose specific restrictions on the type of rolling average used for MMP calculations, which can generally be categorized as simple or weighted. A simple moving average calculates the mean of a fixed number of preceding data points, whereas a weighted moving average applies different weights to data points based on their position in the sequence. Specifically, the exponentially weighted moving average (EWM) uses exponentially decreasing weighting factors, offering several computational advantages. Unlike a simple moving average, EWM can be calculated using a first‐order linear differential equation (Eq. A3), which enables recursive computation, avoiding the need to store full time windows and enabling real‐time implementation (Zignoli and Biral 2024; Zignoli and Whitehurst 2025).

As mentioned in the Supporting Information S1: Appendix A (Eq. A3), the generic EWM model for an individual cycling session can be expressed as follows:

(1) $$\tau_{k}\frac{d{\text{EWM}}_{k}}{\text{dt}}+{\text{EWM}}_{k}=P$$

where *τ*_k_ represents the time constant corresponding to EWM_k_. For practical spreadsheet‐based calculations, the discrete version of this equation is more suitable. At a generic time step *i*, the equation is formulated as follows:

(2) $$\tau_{k}\frac{{\text{EWM}}_{k,i+1}-{\text{EWM}}_{k,i}}{t_{i+1}-t_{i}}+{\text{EWM}}_{k,i}=P_{i}$$

Rearranging, the equation becomes:

(3) $${\text{EWM}}_{k,i+1}=\frac{\left(P_{i}-{\text{EWM}}_{k,i}\right)\cdot \left(t_{i+1}-t_{i}\right)}{\tau_{k}}+{\text{EWM}}_{k,i}$$

Each EWM calculation begins with an initial value of zero (i.e., EWM_k,0_ at *i* = 0). This iterative calculation is applied across all *τ*_k_ values, using recent training session data. For this study, the selected *τ*_k_ values were: 12, 30, 60, 120, 180, 300, 600, 1200, 1800, 3600, 7200, and 14,400 s. Consequently, for each training session, a set of EWM values (EWM_12s_, EWM_30s_, … EWM_14400s_) was calculated. These time constants were log‐spaced to span a wide range of physiological time scales, from rapid fluctuations in power output to more sustained fatigue processes. The spacing between *τ*_k_ values increase with duration, reflecting the fact that performance dynamics evolve more rapidly at short time scales and more gradually over longer durations, as explained in Supporting Information S1: Appendix A. Shorter *τ*_k_ values are therefore more sensitive to rapid changes in power output, whereas longer *τ*_k_ values capture more sustained trends in performance. The interaction between these time scales determines which *τ*_k_ dominates at any given moment.

To construct the historic MMP profile for each cyclist, all training sessions completed during the first 10 days of the training camp were used. For each *τ*_k_, the maximum EWM values (EWM_MAX‐12s_, EWM_MAX‐30s_, … EWM_MAX‐14400s_) were computed. The final “fatiguing” training session was then used to calculate the exercising MMP and validate the methodology.

### 2.3 Maximum Ratio Between Exercising and Previous Maximal Mean Power

For each cyclist, during the final session of the camp (i.e., the session not included in the calculation of EWM_MAX_ values), the ratio between each EWM_k_ and its corresponding historical maxima (EWM_MAX‐_ _*τ*_ _k_) was computed. The maximum ratio, denoted as follows:

(4) $$\max\left\{\frac{{\text{EWM}}_{12s}}{{\text{EWM}}_{\mathrm{MAX}‐12s}},\frac{{\text{EWM}}_{30s}}{{\text{EWM}}_{\mathrm{MAX}‐30s}},...,\frac{{\text{EWM}}_{14400s}}{{\text{EWM}}_{\mathrm{MAX}‐14400s}}\right\}$$

represents the margin relative (%) to the best performance achieved during the first 10 days of the training camp. This formulation defines a set of constraints, where each EWM component is bounded by its historical maximum. The maximum ratio therefore identifies the most critical constraint at each time point. It is important to note that, due to the differential formulation adopted (Equations (1), (2), (3)), each time constant *τ*_k_ is evaluated continuously during the exercise without requiring prior knowledge of the total exercise duration. At each time instant, by computing the ratio between the current power output and its corresponding historical maximum over multiple time scales (*τ*_k_). The maximum of these ratios identifies the dominant time scale at that moment.

The associated *τ*_k_ therefore represents the characteristic time scale governing the current performance state. When the maximum ratio increases toward 100%, the athlete is approaching maximal performance, whereas a decrease toward 0% indicates recovery or distancing from it. In this sense, *τ*_k_ reflects how rapidly the athlete is transitioning toward or away from their performance limit and, possibly, task failure or disengagement from exercise.

### 2.4 Statistical Analysis

All MMP data were checked for normality at each time point with the Shapiro–Wilk normality test. If normality was confirmed, mean and standard deviation (mean (SD)) were used to describe the MMP variables. For time series data, however, median and inter‐quartile range (IQR) were used to represent trends graphically. Similarly, the median and IQR were employed to describe the *τ*_k_ values limiting exercise, given the discrete nature of this variable.

The model's accuracy was evaluated by determining whether the maximum ratio between exercising and previous MMP reached 100% for all cyclists at the end of the 3‐ and 12‐min tests. The model was deemed accurate when the 100% value fell within the IQR at the end of both tests. Paired *t*‐tests with Bonferroni correction were used to compare previous and exercising MMP values at each time point.

All statistical analyses were performed using R (ver. 4.3.3) with custom‐written scripts in RStudio (ver. 2023.12.1). Statistical significance was set at *p* < 0.05.

## 3 Results

The MMP values derived from the maximal EWM across various *τ* values are presented in Table 1 in aggregated form. Normality of distribution was confirmed for all time averages (*p* < 0.001). Paired *t*‐tests comparing the exercising and previous MMP revealed statistically significant differences only for EWM_MAX_ at *τ*_12s_ and *τ*_30s_.

**TABLE 1:** Aggregated maximum power output values (*n* = 21, median and inter‐quartile range) for the exponentially weighted moving average (previous EWM_MAX‐_ _τ_ _k_) across different time constraints (τ: 12 s, 30 s, … 14,400 s).

	Time (s)	Previous EWM_MAX_ (W)	Exercising EWM_MAX_ (W)
*τ*_12s_	12	798 (85)	566 (168)^a^
*τ*_30s_	30	571 (64)	464 (118)^a^
*τ*_60s_	60	477 (37)	437 (108)
*τ*_120s_	120	424 (21)	404 (98)
*τ*_180s_	180	400 (25)	388 (94)
*τ*_300s_	300	378 (26)	364 (88)
*τ*_600s_	600	332 (21)	313 (75)
*τ*_1200s_	1200	287 (18)	272 (64)
*τ*_1800s_	1800	268 (17)	257 (61)
*τ*_3600s_	3600	240 (16)	233 (55)
*τ*_7200s_	7200	203 (16)	194 (47)
*τ*_14400s_	14,400	153 (13)	147 (36)

^a^ Significantly different than previous EWM_MAX‐_ _τ_ _k_ (*p* < 0.001).

**FIGURE 2:**
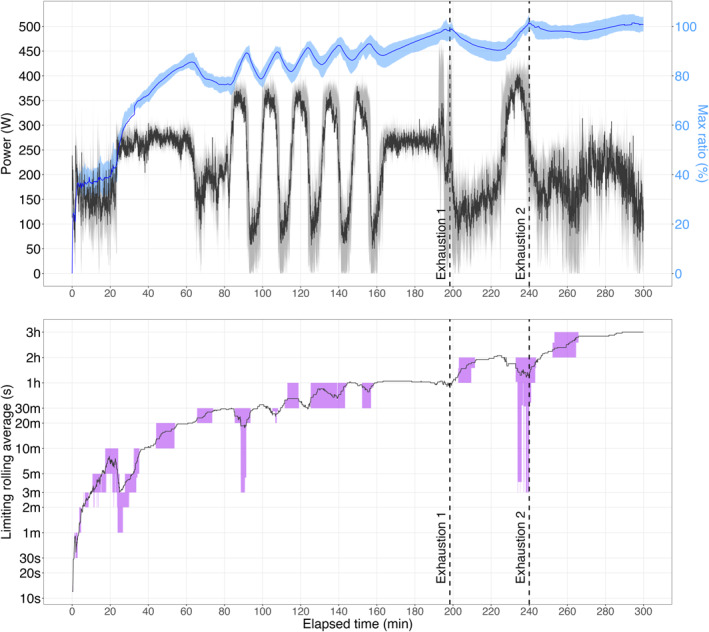
Power output during the fatiguing protocol (TOP graph, in watts, black line) used to compute the ratio between exercising and previous maximal mean power (in %, blue line). The interquartile range is represented by the gray band. The limiting time characteristic (*τ*) is depicted in the BOTTOM graph (log‐transformed y‐axis) with the interquartile range highlighted in purple. Data include all cyclists (*n* = 21). Vertical dashed lines indicate the average times of exhaustion following the first and second bouts.

Figure 2 illustrates the oscillations in the maximum ratio between the exercising and previous MMP, alongside absolute power output values. The corresponding *τ* values associated with the EWM closest to the previous limit are also shown. The maximum ratio between the exercise intensity and prior MMP after the time‐trial protocols were: 98.6% (94.3, 101%) for the 3‐min effort and 101% (98.5, 103%) for the 12‐min effort.

## 4 Discussion

We introduced a new mechanical model with the aim to track progress toward performance limits during prolonged stochastic cycling exercise. As shown in Figure 2, the maximum ratio between the exercise intensity and prior MMP was approximately 100% at the end of the time‐trials: 98.6% (94.3, 101%) for the 3‐min effort and 101% (98.5, 103%) for the 12‐min effort. In both time trials, maximum performance was consistently associated with the depletion of a slow time‐scale component (∼1 h), rather than components corresponding to the nominal duration of the efforts. This suggests that performance limits are determined by exercise history rather than the intensity or duration alone. We suggest that proximity to maximum performance in cycling can be inferred by continuously computing rolling averages of mechanical power output and identifying the dominant component, that is, the component that is approaching its historical maximum at any given moment.

Currently, the most widely adopted models for predicting proximity to maximum performance are the W_bal_ model (Skiba et al. 2012) and their variations (Clarke and Skiba 2013; Skiba et al. 2014; Skiba and Clarke 2021). These models are directly derived from the critical power (CP) framework (Monod and Scherrer 1965; Moritani et al. 1981), which estimates W’ (i.e., the work performed above the CP threshold (Jones and Vanhatalo 2017)) and the CP itself (i.e., the threshold distinguishing heavy from severe exercise intensities (Jones et al. 2010)). Whereas W_bal_ models attempt to describe the body's energy‐production capacity, they do so only within the non‐sustainable intensity domain. However, determining W′ recovery kinetics experimentally often involve tests to “exhaustion” (Bartram et al. 2018; Bourgois et al. 2023; Caen et al. 2021). This practice contradicts the fundamental premise of W_bal_ models, which do not predict “exhaustion” at W′ depletion but instead indicate the point where power output should drop below CP to prevent “exhaustion”. Therefore, given our experimental settings, a direct comparison with W_bal_ models is not straightforward, as the two approaches rely on different modeling assumptions and target different aspects of performance.

In W_bal_ models, it was proposed that during constant workload exercise above CP, W′ is depleted at a rate proportional to the difference between power output and CP, whereas W′ recovery follows a single exponential function (Skiba et al. 2012). This modeling approach assumes that the human body maintains constant efficiency in mechanical energy production. However, not all Joules are created equally. Recent evidence suggests that W′ recovery kinetics depend on prior exercise intensity (Caen et al. 2019) and follow a multi‐exponential pattern (Chorley et al. 2022). However, because of model limitations, (Caen et al. 2021; Chorley et al. 2022) were unable to test recovery behaviors beyond two exponentials, as parameter collinearity made optimization ill‐conditioned (Chorley et al. 2022). To our knowledge, a differential version of a multi‐exponential W_bal_ model has yet to be developed, restricting applicability to step‐input scenarios (see Supporting Information S1: Appendix A).

The model presented here suggests that energy production capacity is depleted and recovered in an exponential manner, as described using EWMs (Supporting Information S1: Appendix A). From a systems perspective, the model can be interpreted as a set of first‐order dynamical states constrained by their historical maxima. Performance limits are reached when at least one of these constraints becomes active. Unlike previous solutions, this model pre‐selects multiple exponentials, enabling the identification of the dominant one—the component that dictates the system's progression toward performance limits. Mathematically, this approach provides an enhanced description of observed performance dynamics and can capture the complexity of performance output across multiple time scales. Furthermore, this approach does not require parameter estimation of recovery kinetics or predefined physiological assumptions.

It has been proposed that the fast exponential of W′ recovery kinetics (*τ* < 20–300 s) may be consistent with physiological processes operating on similar time scales, such as phosphocreatine (PCr) resynthesis, whereas the slow exponential (*τ* ∼ 300–600 s) may be associated with lactate clearance processes (Menzies et al. 2010). (Chorley et al. 2022) observed that W′ recovery slowed after consecutive exercise bouts, with the second bout demonstrating slower W′ recovery. However, numerous studies on W′ recovery (Caen et al. 2021; Felippe et al. 2020; Ferguson et al. 2010) suggest a consensus that very slow recovery processes may be linked to strong aerobic engagement and an elevated aerobic baseline. Even more slowly evolving metabolic processes could be associated with the restoration of acid–base homeostasis, recovery of muscle function and impairment of Ca^2+^ release/sensitivity (Felippe et al. 2020; Ferguson et al. 2010) following prolonged exercise.

**FIGURE 3:**
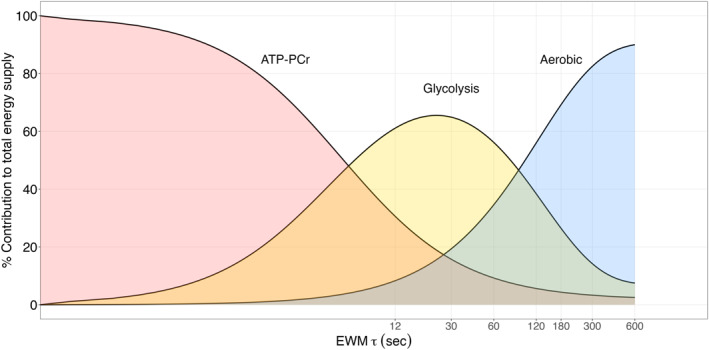
Adapted from Gastin (Gastin 2001): Relative contributions of energy systems to total energy supply, mapped against the kinetic characteristics (*τ*) of the dominant exponentially weighted moving average (EWM), corresponding to the maximal mean power (MMP) closest to its historical maximum. ATP‐PCr refers to the alactic component of the anaerobic energy system. This diagram offers an interpretation of the proposed model and the behavior of the dominant biomarkers by time‐window. If the dominant τ is very short (< 10 s) we expect the phosphagen system and the neural drive/excitation–contraction coupling to be the dominant process, and we would observe somewhat elevated PCr depletion rate and ATP turnover. If the dominant τ is short (10–80 s) we expect anaerobic glycolysis to be the dominant energetic process, and we would observe rapid pH disturbance, elevated blood lactate appearance rate, elevated ventilation and heart rate. If the dominant τ is long (1–10 min) we expect the aerobic metabolism to be the dominant process, and we would observe the appearance of a slow VO_2_ component, drift in heart rate, steady state lactate. If the dominant τ is very long (10–60 min) we expect the aerobic metabolism, the thermoregulation, the central fatigue and the substrate availability to be the dominant limiting systems, alongside the appearance of a drift in heart rate compared to power, and possible rise in core temperature. We expect these biomarkers to become increasingly visible as the ratio of exercising maximal mean power approaches its historical maxima.

Associating recovery kinetics with specific physiological mechanisms oversimplifies the complex and multifactorial nature of fatigue (Abbiss and Laursen 2005; Marcora 2008; Noakes et al. 2005; Tripp et al. 2024). As suggested by (Ferguson et al. 2010) and (Felippe et al. 2020), W′ recovery is influenced not solely by peripheral or central factors, but by the interaction between the two. In addition, reports of W′ recovery kinetics have shown considerable variability (see e.g. (Bartram et al. 2018)) and individuality (Welburn et al. 2026), similar to lactate recovery kinetics (see e.g. (Beneke et al. 2005; Haase et al. 2025; Menzies et al. 2010; Porter and Langley 2025)). With these limitations in mind, we adapted Gastin's (Gastin 2001; Gastin and Suppiah 2026) conceptual diagram for all‐out exercise bouts to represent relative energy system contributions during stochastic exercise. During stochastic exercise, different physiological systems dominate performance and biomarker expression at different time scales, because: A) Energy systems have distinct kinetics, B) regulatory systems have different response times, and C) fatigue accumulates through multiple interacting mechanisms. Therefore, a dominant time exists for each biomarker because their signal‐to‐noise ratio is likely to peak within that time‐window. Therefore, in our adaptations, the x‐axis does not represent exercise duration at constant intensity but rather the time‐characteristics of the dominant exponential (i.e., the MMP closest to the historical maximum), and the different metabolic pathways associated (Figure 3). Importantly, this diagram should not be interpreted as indicating discrete “all‐or‐nothing” mechanisms but rather as reflecting the interplay of multiple pathways contributing to energy production. Indeed, energy metabolism, encompassing both anaerobic and aerobic components, operates as a dynamic interplay of coordinated fluxes rather than discrete systems (Martínez‐Reyes and Chandel 2017). These fluxes are influenced by systemic factors, such as the autonomic nervous system, and local factors, including substrate availability and metabolic capacity (Hargreaves and Spriet 2020).

The diagram in Figure 3 provides a framework for interpreting this model features and generating hypotheses for future research. A key finding of this study is that the dominant time scale governing performance limits shifts depending on prior load, with prolonged exercise favoring slower components. Results indicate that performance limits at the end of both time trials were not driven by the depletion of exponentials corresponding to the trial durations (i.e., 3 and 12 min). Instead, at the limits of the performance, the dominant exponential component was much slower (∼1 h, Figure 2). This suggests that in a fresh state, shorter (faster) exponentials would likely govern maximum performance during 3‐ and 12‐min time trials. According to this model, a fresh state would favor glycolytic/anaerobic pathways, leading to high lactate responses and rapid PCr recovery. Conversely, in a fatigued state, the same time trials would rely more on aerobic pathways, marked by oxygen drift, elevated heart rate, and reduced efficiency (Stevenson et al. 2022). In essence, this model suggests a potential shift toward a greater fractional aerobic contribution to total energy turnover in a fatigued state, despite similar power and duration. Although experimental data are needed to validate this, recent research on exercise “durability” support this hypothesis, showing reduced anaerobic metabolism following prolonged cycling (Gallo et al. 2024; Ørtenblad et al. 2024).

This model has inherent limitations, primarily its reliance on historical data for predictions. In competitive settings, actual performance may surpass predicted capacities if prior data fail to capture peak efforts. If an athlete exceeds previously recorded maxima, ratios may transiently exceed 100%, indicating the need to update the historical profile. Additionally, the model does not account for environmental factors such as temperature and humidity (Valenzuela et al. 2022), internal factors such as metabolic capacity and substrate availability, hydration status, motivation, or sleep quality. Also, this model only takes into accounts the best efforts irrespective of bike position, for example time‐trial/aerodynamic versus standard (Fintelman et al. 2014; Kordi et al. 2019). Another limitation is the exclusive use of absolute power values to construct the MMP profile; incorporating a weight‐adjusted metric (e.g., W/kg) could enhance prediction accuracy. Despite the model's high accuracy in predicting performance limits, real‐world outdoor data collection presents challenges (measurement noise and device variability may influence EWM estimates, particularly at shorter time scales). Synchronizing efforts across cyclists may have introduced minor inaccuracies in the aggregated estimates. Perhaps the greatest limitation is theoretical. Although this model aids in interpreting the physiological effects of prolonged exercise (Maunder et al. 2021), it remains a mechanical model. It does not directly simulate physiological processes but rather their observable manifestations by means of a mechanical output.

**FIGURE 4:**
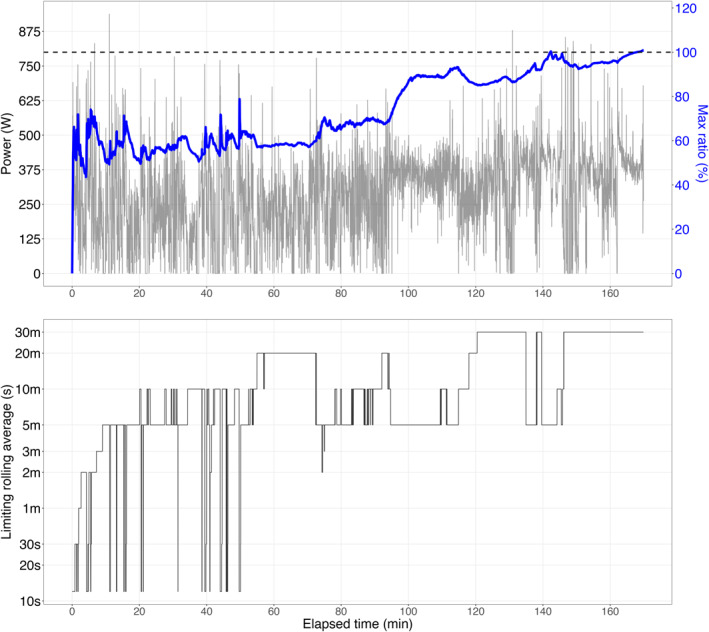
Power output during a climbing stage for a single professional cyclist involved in a Grand Tour stage. The ratio between exercising and previous maximal mean power (in %, blue line) is plotted alongside the power output (gray line) (UPPER GRAPH). The kinetics of the dominant exponential (*τ*) is illustrated in the BOTTOM GRAPH (log‐transformed y‐axis).

Although the session protocol employed in this validation study was unique, we believe that the findings are generalizable and applicable across various session protocols. Two additional practical applications are provided in Supporting Information S1: Appendix B to illustrate real‐world use cases (see Figures 4 and 5 in the Supporting Information S1: Appendix). Also, although this methodology was validated in a cycling context, it is broadly applicable to other forms of continuous exercise, such as running, swimming, rowing, and even team sports (Cassirame et al. 2022). The model's computational efficiency, achieved through the discrete formulation of ordinary differential equations (Equation 3), facilitates real‐time applications on lightweight hardware, such as cycling computers or running watches. For example, the model has been deployed on a Garmin Connect IQ app and is used to monitor their MMP ratios in real time (apps.garmin.com) (Zignoli and Whitehurst 2025). Furthermore, the model has demonstrated potential in optimizing pacing strategy (Zignoli and Biral 2024) and prescribing training sessions, enabling rapid simulations of virtual sessions to evaluate sustainability based on an athlete's MMP.

**FIGURE 5:**
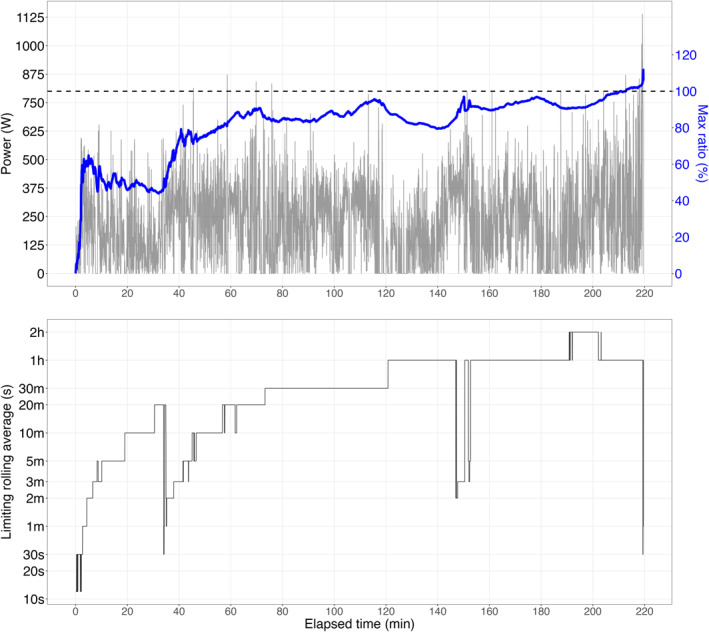
Power output during a sprint stage for a single professional cyclist involved in a multi‐stage race. The ratio between exercising and previous maximal mean power (in %, blue line) is plotted alongside the power output (gray line) (UPPER GRAPH). The kinetics of the dominant exponential (*τ*) is depicted in the BOTTOM GRAPH (log‐transformed y‐axis).

In conclusion, this work introduces a multi‐timescale dynamical framework in which proximity to maximum performance emerges from the interaction between current power output and historically observed limits. By identifying the dominant time scale governing performance at each moment, the model provides new insight into how prior load shapes endurance capacity. We propose that: (1) Margins to performance limits can be tracked with the dominant component of the MMP, and (2) performance limits are influenced not only by effort intensity and duration but also by the cumulative effects of prior intensity variations. This framework enables the assessment of whether an athlete is transitioning toward or away from their performance limits and at what rate their remaining performance capacity is depleting or recovering.

## Funding

The authors have nothing to report.

## Conflicts of Interest

A.Z., P.B.L. and F.K. hold stocks in Athletica (https://athletica.ai/), an online training platform that implements the model introduced under the name “Athletica Workout Reserve (TM)”. The model is also available as Garmin IQ app (https://apps.garmin.com/apps/c9a93545‐7db0‐4a1b‐b955‐21db19edbf9d?tid=2) for real‐time monitoring of Workout Reserve in relation to maximal mean power (cycling) or speed (running). Access to the real‐time app requires signing up for a free trial or a monthly/yearly subscription to the Athletica platform. This platform is critical for constructing and synchronizing the power/speed profiles before training or racing. To safeguard the company's intellectual property, a slightly modified version of the model is implemented on Athletica, with specific details remaining confidential.

## Supporting information


Supporting Information S1

## Data Availability

The data that support the findings of this study are available from the corresponding author upon reasonable request.

## References

[ejsc70179-bib-0001] Abbiss, C. R. , and P. B. Laursen . 2005. “Models to Explain Fatigue During Prolonged Endurance Cycling.” Sports Medicine 35, no. 10: 865–898. 10.2165/00007256-200535100-00004. 16180946

[ejsc70179-bib-0002] Bartram, J. C. , D. Thewlis , D. T. Martin , and K. I. Norton . 2018. “Accuracy of W′ Recovery Kinetics in High Performance Cyclists—Modeling Intermittent Work Capacity.” International Journal of Sports Physiology and Performance 13, no. 6: 724–728. 10.1123/ijspp.2017-0034. 29035607

[ejsc70179-bib-0003] Beneke, R. , M. Hütler , M. Jung , and R. M. Leithäuser . 2005. “Modeling the Blood Lactate Kinetics at Maximal Short‐Term Exercise Conditions in Children, Adolescents, and Adults.” Journal of Applied Physiology 99, no. 2: 499–504. 10.1152/japplphysiol.00062.2005. 16020438

[ejsc70179-bib-0004] Bourgois, G. , P. Mucci , J. Boone , et al. 2023. “Critical Power, W′ and W′ Reconstitution in Women and Men.” European Journal of Applied Physiology 123, no. 12: 2791–2801. 10.1007/s00421-023-05268-3. 37369796

[ejsc70179-bib-0005] Caen, K. , G. Bourgois , C. Dauwe , et al. 2021. “W′ Recovery Kinetics After Exhaustion: A Two‐phase Exponential Process Influenced by Aerobic Fitness.” Medicine & Science in Sports & Exercise 53, no. 9: 1911–1921. 10.1249/MSS.0000000000002673. 33787532

[ejsc70179-bib-0006] Caen, K. , J. G. Bourgois , G. Bourgois , T. Van Der Stede , K. Vermeire , and J. Boone . 2019. “The Reconstitution of W′ Depends on Both Work and Recovery Characteristics.” Medicine & Science in Sports & Exercise 51, no. 8: 1745–1751. 10.1249/MSS.0000000000001968. 31083026

[ejsc70179-bib-0007] Cassirame, J. , M. Coulerot , C. Manouvrier , C. Osgnach , and P. E. di Prampero . 2022. “Metabolic Power Profile in Soccer Based on GPS Measurement Concept and Caveats.” Sport Performance & Science Reports: 116. https://www.researchgate.net/publication/364334330_Metabolic_power_profile_in_soccer_based_on_GPS_measurement_concept_and_caveats.

[ejsc70179-bib-0008] Chorley, A. , R. P. Bott , S. Marwood , and K. L. Lamb . 2022. “Bi‐Exponential Modelling of W’ Reconstitution Kinetics in Trained Cyclists.” European Journal of Applied Physiology 122, no. 3: 677–689. 10.1007/s00421-021-04874-3. 34921345 PMC8854279

[ejsc70179-bib-0009] Clarke, D. C. , and P. F. Skiba . 2013. “Rationale and Resources for Teaching the Mathematical Modeling of Athletic Training and Performance.” Advances in Physiology Education 37, no. 2: 134–152. 10.1152/advan.00078.2011. 23728131

[ejsc70179-bib-0010] Felippe, L. C. , T. G. Melo , M. D. Silva‐Cavalcante , et al. 2020. “Relationship Between Recovery of Neuromuscular Function and Subsequent Capacity to Work Above Critical Power.” European Journal of Applied Physiology 120, no. 6: 1237–1249. 10.1007/s00421-020-04338-0. 32318812

[ejsc70179-bib-0011] Ferguson, C. , H. B. Rossiter , B. J. Whipp , A. J. Cathcart , S. R. Murgatroyd , and S. A. Ward . 2010. “Effect of Recovery Duration From Prior Exhaustive Exercise on the Parameters of the Power‐Duration Relationship.” Journal of Applied Physiology 108, no. 4: 866–874. 10.1152/japplphysiol.91425.2008. 20093659

[ejsc70179-bib-0012] Fintelman, D. M. , M. Sterling , H. Hemida , and F.‐X. Li . 2014. “Optimal Cycling Time Trial Position Models: Aerodynamics Versus Power Output and Metabolic Energy.” Journal of Biomechanics 47, no. 8: 1894–1898. 10.1016/j.jbiomech.2014.02.029. 24726654

[ejsc70179-bib-0013] Gallo, G. , E. L. Faelli , P. Ruggeri , et al. 2024. “Power Output at the Moderate‐to‐Heavy Intensity Transition Decreases in a Non‐linear Fashion During Prolonged Exercise.” European Journal of Applied Physiology 124, no. 8: 2353–2364. 10.1007/s00421-024-05440-3. 38483635 PMC11322563

[ejsc70179-bib-0014] Gastin, P. B. 2001. “Energy System Interaction and Relative Contribution During Maximal Exercise.” Sports Medicine 31, no. 10: 725–741. 10.2165/00007256-200131100-00003. 11547894

[ejsc70179-bib-0015] Gastin, P. B. , and H. T. Suppiah . 2026. “Anaerobic and Aerobic Energy System Contribution During Maximal Exercise: A Systematic Review.” Sports Medicine. 10.1007/s40279-026-02414-7. PMC13388521 41965479

[ejsc70179-bib-0016] Haase, R. , A. K. Dunst , and N. Nitzsche . 2025. “Blood Lactate Accumulation During Maximal Cycling Sprints and Its Relationship to Sprint Performance Characteristics.” European Journal of Applied Physiology 125, no. 8: 2197–2207. 10.1007/s00421-025-05755-9. 40111462 PMC12354552

[ejsc70179-bib-0017] Hargreaves, M. , and L. L. Spriet . 2020. “Skeletal Muscle Energy Metabolism During Exercise.” Nature Metabolism 2, no. 9: 817–828. 10.1038/s42255-020-0251-4. 32747792

[ejsc70179-bib-0018] Jobson, S. A. , L. Passfield , G. Atkinson , G. Barton , and P. Scarf . 2009. “The Analysis and Utilization of Cycling Training Data.” Sports Medicine 39, no. 10: 833–844. 10.2165/11317840-000000000-00000. 19757861

[ejsc70179-bib-0019] Jones, A. M. , and A. Vanhatalo . 2017. “The ‘Critical Power’ Concept: Applications to Sports Performance With a Focus on Intermittent High‐Intensity Exercise.” Supplement, Sports Medicine 47, no. S1: 65–78. 10.1007/s40279-017-0688-0. 28332113 PMC5371646

[ejsc70179-bib-0020] Jones, A. M. , A. Vanhatalo , M. Burnley , R. H. Morton , and D. C. Poole . 2010. “Critical Power: Implications for Determination of V˙O2max and Exercise Tolerance.” Medicine & Science in Sports & Exercise 42, no. 10: 1876–1890. 10.1249/MSS.0b013e3181d9cf7f. 20195180

[ejsc70179-bib-0021] Kordi, M. , C. Fullerton , L. Passfield , and L. Parker Simpson . 2019. “Influence of Upright Versus Time Trial Cycling Position on Determination of Critical Power and W′ in Trained Cyclists.” European Journal of Sport Science 19, no. 2: 192–198. 10.1080/17461391.2018.1495768. 30009673

[ejsc70179-bib-0022] Leo, P. , J. Spragg , T. Podlogar , J. S. Lawley , and I. Mujika . 2022. “Power Profiling and the Power‐Duration Relationship in Cycling: A Narrative Review.” European Journal of Applied Physiology 122, no. 2: 301–316. 10.1007/s00421-021-04833-y. 34708276 PMC8783871

[ejsc70179-bib-0023] Marcora, S. M. 2008. “Do We Really Need a Central Governor to Explain Brain Regulation of Exercise Performance?” European Journal of Applied Physiology 104, no. 5: 929–931. 10.1007/s00421-008-0818-3. 18618133

[ejsc70179-bib-0024] Martínez‐Reyes, I. , and N. S. Chandel . 2017. “Waste Not, Want Not: Lactate Oxidation Fuels the TCA Cycle.” Cell Metabolism 26, no. 6: 803–804. 10.1016/j.cmet.2017.11.005. 29211977

[ejsc70179-bib-0025] Mateo‐March, M. , P. L. Valenzuela , X. Muriel , et al. 2022. “The Record Power Profile of Male Professional Cyclists: Fatigue Matters.” International Journal of Sports Physiology and Performance 17, no. 6: 926–931. 10.1123/ijspp.2021-0403. 35240578

[ejsc70179-bib-0026] Maunder, E. , S. Seiler , M. J. Mildenhall , A. E. Kilding , and D. J. Plews . 2021. “The Importance of ‘Durability’ in the Physiological Profiling of Endurance Athletes.” Sports Medicine 51, no. 8: 1619–1628. 10.1007/s40279-021-01459-0. 33886100

[ejsc70179-bib-0027] McCormick, A. , C. Meijen , and S. Marcora . 2015. “Psychological Determinants of Whole‐Body Endurance Performance.” Sports Medicine 45, no. 7: 997–1015. 10.1007/s40279-015-0319-6. 25771784 PMC4473096

[ejsc70179-bib-0028] Menzies, P. , C. Menzies , L. McIntyre , P. Paterson , J. Wilson , and O. J. Kemi . 2010. “Blood Lactate Clearance During Active Recovery After an Intense Running Bout Depends on the Intensity of the Active Recovery.” Journal of Sports Science 28, no. 9: 975–982. 10.1080/02640414.2010.481721. 20544484

[ejsc70179-bib-0029] Monod, H. , and J. Scherrer . 1965. “The Work Capacity of a Synergic Muscular Group.” Ergonomics 8, no. 3: 329–338. 10.1080/00140136508930810.

[ejsc70179-bib-0030] Moritani, T. , A. Nagata , H. Devries , and M. Muro . 1981. “Critical Power as a Measure of Physical Work Capacity and Anaerobic Threshold.” Ergonomics 24, no. 5: 339–350. 10.1080/00140138108924856. 7262059

[ejsc70179-bib-0031] Noakes, T. D. , A. St Clair Gibson , and E. V. Lambert . 2005. “From Catastrophe to Complexity: A Novel Model of Integrative Central Neural Regulation of Effort and Fatigue During Exercise in Humans: Summary and Conclusions.” British Journal of Sports Medicine 39, no. 2: 120–124. 10.1136/bjsm.2003.010330. 15665213 PMC1725112

[ejsc70179-bib-0032] Ørtenblad, N. , M. Zachariassen , J. Nielsen , and K. D. Gejl . 2024. “Substrate Utilization and Durability During Prolonged Intermittent Exercise in Elite Road Cyclists.” European Journal of Applied Physiology 124, no. 7: 2193–2205. 10.1007/s00421-024-05437-y. 38441690 PMC11199313

[ejsc70179-bib-0033] Pinot, J. , and F. Grappe . 2011. “The Record Power Profile to Assess Performance in Elite Cyclists.” International Journal of Sports Medicine 32, no. 11: 839–844. 10.1055/s-0031-1279773. 22052032

[ejsc70179-bib-0034] Porter, M. , and J. Langley . 2025. “The Relationship Between Muscle Oxygen Saturation Kinetics and Maximal Blood Lactate Accumulation Rate Across Varying Sprint Cycle Durations.” European Journal of Sport Science 25, no. 3: e12242. 10.1002/ejsc.12242. 40017007 PMC11868032

[ejsc70179-bib-0035] Quod, M. J. , D. T. Martin , J. C. Martin , and P. B. Laursen . 2010. “The Power Profile Predicts Road Cycling MMP.” Int J Sports Med 31, no. 6: 397–401. 10.1055/s-0030-1247528. 20301046

[ejsc70179-bib-0036] Skiba, P. F. , W. Chidnok , A. Vanhatalo , and A. M. Jones . 2012. “Modeling the Expenditure and Reconstitution of Work Capacity Above Critical Power.” Medicine & Science in Sports & Exercise 44, no. 8: 1526–1532. 10.1249/mss.0b013e3182517a80. 22382171

[ejsc70179-bib-0037] Skiba, P. F. , D. Clarke , A. Vanhatalo , and A. M. Jones . 2014. “Validation of a Novel Intermittent W’Model for Cycling Using Field Data.” International Journal of Sports Physiology and Performance 9, no. 6: 900–904. 10.1123/ijspp.2013-0471. 24509723

[ejsc70179-bib-0038] Skiba, P. F. , and D. C. Clarke . 2021. “The W′ Balance Model: Mathematical and Methodological Considerations.” International Journal of Sports Physiology and Performance 16, no. 11: 1561–1572. 10.1123/ijspp.2021-0205. 34686611

[ejsc70179-bib-0039] Spragg, J. , P. Leo , A. Giorgi , B. M. Gonzalez , and J. Swart . 2024. “The Intensity Rather Than the Quantity of Prior Work Determines the Subsequent Downward Shift in the Power Duration Relationship in Professional Cyclists.” European Journal of Sport Science 12077, no. 4: 449–457. 10.1002/ejsc.12077.

[ejsc70179-bib-0040] Stevenson, J. D. , A. E. Kilding , D. J. Plews , and E. Maunder . 2022. “Prolonged Cycling Reduces Power Output at the Moderate‐to‐Heavy Intensity Transition.” European Journal of Applied Physiology 122, no. 12: 2673–2682. 10.1007/s00421-022-05036-9. 36127418 PMC9488873

[ejsc70179-bib-0041] Tripp, T. R. , A. M. Caswell , S. J. Aboodarda , and M. J. MacInnis . 2024. “The Effect of Duration on Performance and Perceived Fatigability During Acute High‐Intensity Interval Exercise in Young, Healthy Males and Females.” Scandinavian Journal of Medicine & Science in Sports 34, no. 7: e14692. 10.1111/sms.14692. 38982705

[ejsc70179-bib-0042] Valenzuela, P. L. , M. Mateo‐March , X. Muriel , et al. 2023. “Between‐Seasons Variability of Cyclists’ Peak Performance: A Longitudinal Analysis of “Real‐World” Power Output Data in Male Professional Cyclists.” International Journal of Sports Physiology and Performance 18, no. 10: 1141–1144. 10.1123/ijspp.2023-0042. 37385604

[ejsc70179-bib-0043] Valenzuela, P. L. , M. Mateo‐March , M. Zabala , et al. 2022. “Ambient Temperature and Field‐Based Cycling Performance: Insights From Male and Female Professional Cyclists.” International Journal of Sports Physiology and Performance 17, no. 7: 1025–1029. 10.1123/ijspp.2021-0508. 35338106

[ejsc70179-bib-0044] Welburn, A. J. , C. F. Pugh , S. J. Bailey , and R. A. Ferguson . 2026. “W′ Reconstitution Modelling During Intermittent Exercise Performed to Task Failure.” European Journal of Applied Physiology 126, no. 2: 765–778. 10.1007/s00421-025-05912-0. 40788399 PMC12948861

[ejsc70179-bib-0045] Zignoli, A. , and F. Biral . 2024. “Incorporating the Maximal Mean Power Profile in Time Trial Simulations for More Efficient Optimal Pacing Strategy Calculations.” Journal of Science and Cycling 13: 10–12. https://www.jsc‐journal.com/index.php/JSC/article/view/932.

[ejsc70179-bib-0046] Zignoli, A. , and P. Whitehurst . 2025. “Real‐Time Assessment of Exercising Maximal Mean Power and Speed in Endurance Sports: A Garmin Connect IQ App.” Sports Engineering 28, no. 2: 45. 10.1007/s12283-025-00528-1.

