# Supplementary material for: Tracking Performance Limits Using Multi‐Timescale Maximal Mean Power Ratios
Source: Eur J Sport Sci. 2026 May 18;26(6):e70179. doi: 10.1002/ejsc.70179 (PMC13181598; doi:10.1002/ejsc.70179)
Supplement: Supplementary file 1 — Supporting Information S1 [file EJSC-26-e70179-s001.docx]

**Appendix A**

Mathematical models are widely used across various scientific disciplines, often sharing the same foundational equations. A notable example demonstrates a framework with broad applicability, ranging from physics to exercise physiology. As a first application, consider the equation used to model the voltage (V_C_) of a resistance-capacitor (RC) electrical circuit in response to a varying voltage source (V_IN_):

| $\tau\frac{dV_{C}}{\mathrm{dt}}+V_{C}=V_{IN}$ | (Eq. A1) |
| --- | --- |

Here, 𝛕 is the time constant, defined as the product of the capacitor’s capacitance (C, in Farads) and the resistance (R, in Ohms). The operator d/dt represents the time derivative. This equation is familiar to exercise physiologists (Jones and Poole, 2013) as it is commonly used to describe oxygen consumption ($\dot{V}$O_2_, in mlO_2_/min) at the onset of submaximal exercise:

| $\tau\frac{d{\dot{V}O}_{2}}{\mathrm{dt}}+{\dot{V}O}_{2}=A$ | (Eq. A2) |
| --- | --- |

In this context, A represents the target oxygen uptake determined by the metabolic demand of a specific exercise intensity, and 𝛕 (in seconds) characterizes the $\dot{V}$O_2_ kinetics (Jones and Poole, 2013).

Remarkably, **Eq. A1** and **A2** resemble those used to calculate the exponentially weighted moving average (EWM) of a generic signal y:

| $\tau\frac{\mathrm{dEWM}}{\mathrm{dt}}+\mathrm{EWM}=y$ | (Eq. A3) |
| --- | --- |

Here, 𝛕 (in seconds) represents the span or half-life of the EWM. This formulation, originally introduced in the mid-20th century in economics to monitor stock prices (Roberts, 1959), offers broad applicability.

Models governed by identical equations exhibit equivalent responses when subjected to the same inputs^[[1]](#footnote-2)^. For a step input, the response can be described by a straightforward analytical expression:

| $V_{C}=V_{IN}\left( 1-e^{-t/\tau} \right)$ | (Eq. A4a) |
| --- | --- |
| ${\dot{V}O}_{2}=A\left( 1-e^{-t/\tau} \right)$ | (Eq. A4b) |
| $EWM=y\left( 1-e^{-t/\tau} \right)$ | (Eq. A4c) |

In **Eqs. A1-A3**, the role of V_IN_, A, and y are analogous: they determine the steady-state value toward which VC, $\dot{V}$O_2_, and EWM converge. Similarly, 𝛕 consistently quantifies the rate at which the system responds to input variations–the smaller the 𝛕, the faster the response.

This equation also applies to bioenergetic models of exercise, which aim to elucidate energy source fluctuations and metabolic pathway involvement. In bioenergetic models derived from the critical power (CP) model, such as the W_bal_ models (Skiba and Clarke, 2021), the recovery of W' (i.e., the work capacity above CP (Jones and Vanhatalo, 2017)) is similarly described using an exponential equation akin to a first-order differential equation:

| $\tau\frac{dW'}{\mathrm{dt}}={(CP-P)}^{+}$ | (Eq. A5) |
| --- | --- |

This equation illustrates that, for power outputs (P) below the critical power CP, the restoration rate of W' is governed by 𝛕 and is proportional to the difference between CP and P, as illustrated in **Eq. A4a-A4c**). Notably, distinct values of 𝛕 can be applied to W’ depletion and recovery phases.

Modelling choices reflect the behavior of the underlying system or our understanding of it. Models are often limited in their applicability to specific conditions. For instance, the single-exponential $\dot{V}$O_2_ model represents only the initial fast component of $\dot{V}$O_2_ kinetics (Andrew M Jones et al., 2010). Similarly, W_bal_ models have well-documented constraints (see main text).

The practice of combining multiple exponential behaviors is well-established. Clarke and Skiba (Clarke and Skiba, 2013) provide a clear explanation of this concept, drawing from Banister et al. (Banister et al., 1975), who used the same differential equations in their impulse-response model to distinguish acute negative effects from chronic positive effects of training loads on performance capacity. In Banister’s model (Banister et al., 1975), acute and chronic training loads are described by first-order linear differential equations with different time constraints (𝛕=1 week for acute effects and 𝛕=6 weeks for chronic effects). This demonstrates how combining multiple equations can provide a more realistic representation of complex systems.

Following this reasoning, a single equation (e.g., **Eq. A5**) may be insufficient to capture the intricate dynamics of metabolic pathways during exercise. This assumption is supported by two key observations. First, brief efforts do not necessarily equate to high intensity, nor do they consistently suggest dominant anaerobic metabolism. For example, metabolic inertia at the onset of exercise can elicit substantial anaerobic activity even at low mechanical outputs, as evidenced by $\dot{V}$O_2_ kinetics (Andrew M Jones et al., 2010). Second, assessing exercise intensity without accounting for duration provides an incomplete picture, as recovery rates vary depending on prior work accumulation and the metabolic pathways engaged.

**Appendix B**

Two real-world use cases are presented here involving two professional cyclists. Both cyclists contributed to this study by providing data from their training camp and are members of the same professional cycling team (see **Methods** in the main text).

In these examples, G.P. (21 years old, 1.83 m, 66 kg) and E.Z. (26 years old, 1.70 m, 64 kg) provided six weeks of training and racing data, which were used to construct their power profiles. Their racing data, however, was excluded from the profile construction and utilized solely for post-processing analysis. Both cyclists delivered all-out efforts during key race stages: G.P. in a climbing stage (16th stage of the Giro d’Italia 2024, a 2.UWT class race, where he finished 2nd overall) and E.Z. in a sprint stage (1st stage of the Giro d’Abruzzo 2024, a 2.1 class race, where he achieved 1st overall).

The new model proposed in this manuscript was used to compute the ratio between the current exercise power and the previously established maximal mean power, as well as to identify the kinetics of the dominant exponential (𝞃) leading to performance limits. In both cases, the ratio between the exercise power and historical maximal mean power reached 100%, indicating maximum effort and a peak in exercise capacity.

In the first case, during the climbing stage (**Fig. 4A**), the maximum mean power for a 5-minute duration reached 100% during a breakaway attempt, as documented through live images. In the second case, during the sprint stage (**Fig. 5A**), the maximum mean power for a 30-second duration reached and exceeded 100% during the final sprint.

**<<<< Figure 4A about here >>>>**

**<<<< Figure 5A about here >>>>**

1. All systems with this mathematical structure function as low-pass filters, meaning they reduce the amplitude of input signals with a frequency above a specific cut-off frequency (f_cut-off_):

   | $f_{cut-off}=\frac{1}{\sqrt{2\pi\tau}}$ |  |
   | --- | --- |

   Therefore, by adjusting the capacitance of the RC circuit, either by changing capacitance values or the distance between capacitor plates, one can achieve varying time characteristics and cut-off frequencies. Similarly, individuals with lower aerobic fitness experience a slower increase in $\dot{V}$O_2_ at the onset of exercise, resulting in a larger $\dot{V}$O_2_ time characteristic compared with well-trained individuals (Koppo et al., 2004). [↑](#footnote-ref-2)
